# Mutation of L-2,3-diaminopropionic acid synthase genes blocks staphyloferrin B synthesis in *Staphylococcus aureus*

**DOI:** 10.1186/1471-2180-11-199

**Published:** 2011-09-09

**Authors:** Federico C Beasley, Johnson Cheung, David E Heinrichs

**Affiliations:** 1 Department of Microbiology & Immunology, University of Western Ontario, 1151 Richmond Street, London, Ontario, N6A 5C1, Canada; 2 Centre for Human Immunology, University of Western Ontario, 1151 Richmond Street, London, Ontario, N6A 5C1, Canada

## Abstract

**Background:**

*Staphylococcus aureus *synthesizes two siderophores, staphyloferrin A and staphyloferrin B, that promote iron-restricted growth. Previous work on the biosynthesis of staphyloferrin B has focused on the role of the synthetase enzymes, encoded from within the *sbnA-I *operon, which build the siderophore from the precursor molecules citrate, alpha-ketoglutarate and L-2,3-diaminopropionic acid. However, no information yet exists on several other enzymes, expressed from the biosynthetic cluster, that are thought to be involved in the synthesis of the precursors (or synthetase substrates) themselves.

**Results:**

Using mutants carrying insertions in *sbnA *and *sbnB*, we show that these two genes are essential for the synthesis of staphyloferrin B, and that supplementation of the growth medium with L-2,3-diaminopropionic acid can bypass the block in staphyloferrin B synthesis displayed by the mutants. Several mechanisms are proposed for how the enzymes SbnA, with similarity to cysteine synthase enzymes, and SbnB, with similarity to amino acid dehydrogenases and ornithine cyclodeaminases, function together in the synthesis of this unusual nonproteinogenic amino acid L-2,3-diaminopropionic acid.

**Conclusions:**

Mutation of either *sbnA *or *sbnB *result in abrogation of synthesis of staphyloferrin B, a siderophore that contributes to iron-restricted growth of *S. aureus*. The loss of staphyloferrin B synthesis is due to an inability to synthesize the unusual amino acid L-2,3-diaminopropionic acid which is an important, iron-liganding component of the siderophore structure. It is proposed that SbnA and SbnB function together as an L-Dap synthase in the *S. aureus *cell.

## Background

The stimulus of iron limitation is a key sensory trigger for virtually all bacteria. Iron is scarce in the fluids of mammalian hosts, due to rapid oxidation of the soluble ferrous (Fe^2+^) form to the less soluble ferric (Fe^3+^) form under aerobic, physiological conditions [1]. Any residual soluble ferric iron is further sequestered through high affinity binding by innate immune proteins such as lactoferrin and transferrin [2]. For many pathogenic microbes, decreasing iron availability leads to the enhanced expression of iron acquisition mechanisms and virulence factors, which frequently play direct roles in liberating iron from host sequestration factors [2-4]. A prevalent component of bacterial iron responses is the secretion of siderophores. These small molecules scavenge residual ferric iron as well as transferrin-bound iron from the extracellular milieu with extremely high affinity and are actively reimported into bacterial cells via dedicated ABC-type transport systems [5,6]. Siderophore assembly pathways fall into two broad classes: nonribosomal peptide synthesis (NRPS) and NRPS-independent siderophore (NIS) synthesis [7,8]. NRPS siderophores are peptidic constructs assembled in a stepwise fashion by large, heterofunctional, multidomain proteins, independently of ribosomes. NIS siderophores are formed via condensation of alternating subunits of dicarboxylic acids with diamines, amino alcohols, and alcohols by sets of synthetase enzymes.

Encoded within the genome of *S. aureus *are two loci directing the production of NIS-type siderophores. The *sfaABCD *locus encodes for proteins involved in biosynthesis and secretion of staphyloferrin A, a molecule also produced by the majority of less pathogenic coagulase-negative staphylococci [9-12]. This metabolite is assembled from one unit of the nonproteinogenic amino acid D-ornithine and two units of citrate; the staphyloferrin A biosynthetic pathway was recently established in an elegant study [10]. The *sbnABCDEFGHI *operon encodes for biosynthesis and secretion of staphyloferrin B. This siderophore has been identified in *S. aureus *and a few species of coagulase-negative staphylococci, and in the Gram-negative genera *Ralstonia *and *Cupriavidus *[13-16]. However, based on early studies by Haag *et al*. [16] and recent staphylococcal genome data, staphyloferrin B may also be produced by other coagulase-positive staphylococci other than *S. aureus*. Staphyloferrin B is comprised of one unit each of citric acid, 1,2-diaminoethane, alpha-ketoglutaric acid, and the nonproteinogenic amino acid L-2,3-diaminopropionic acid (L-Dap) [15-17]. These precursors are condensed by NIS synthetase enzymes SbnC, SbnE, and SbnF, with modification of an intermediate metabolite by decarboxylase SbnH [17]. Inactivation of staphyloferrin B biosynthesis (via chromosomal deletion of a siderophore synthetase) was previously shown to reduce the virulence of *S. aureus *in a mouse infection model [14], which underscores the contribution of specialized iron uptake mechanisms to pathogenesis. In addition to the characterized functions of SbnCEFH in staphyloferrin B biosynthesis, the *sbn *operon also encodes several auxiliary enzymes predicted to be involved in generation of staphyloferrin B precursors or substrates that would be fed to the NIS synthetases. Of interest are the first two genes *sbnA *and *sbnB*, which encode proteins with a yet undiscovered role in staphyloferrin B biosynthesis. Furthermore, it is intriguing that SbnA and SbnB share sequence homology to the enzymes VioB and VioK, respectively, of the viomycin assembly pathway in *Streptomyces sp*. [18]. Like staphyloferrin B, the antibiotic viomycin molecule also contains L-Dap as a structural component. It was hypothesized by Thomas *et al*. [18] that VioB (homologous to SbnA) catalyzes a β-substitution replacement reaction to generate L-Dap from (O-acetyl-)L-serine using ammonia as a nucleophile. The source of this ammonia would come from the activity of VioK, which like SbnB, shares sequence identity with bacterial ornithine cyclodeaminases that would catalyze the cyclization of L-Orn to L-Pro with concomitant release of ammonia. Therefore, it is probable that VioK and VioB (or SbnA and SbnB) function synergistically as an L-Dap synthase. The production of L-Dap is a critical process because the molecule is used twice per mole of staphyloferrin B [17]. Specifically, both prochiral carboxyl groups of citrate are condensed onto a molecule of L-Dap as catalyzed by the synthetases SbnE and SbnF [17].

In this study, through a series of genetics-based experiments, we propose that the generation of L-Dap in *S. aureus *is a coupled function of enzymes SbnA and SbnB, whose activity is essential for the downstream biosynthesis of the siderophore staphyloferrin B.

## Methods

### Strains and growth conditions

Bacterial strains, plasmids and oligonucleotides used throughout the study are described in Table 1. *E. coli *strains were grown in Luria-Bertani broth, with the following antibiotic concentrations used for selection of plasmids: kanamycin (30 μg/mL), ampicillin (100 μg/mL), erythromycin (300 μg/mL). *S. aureus *strains were grown in tryptic soy broth for genetic manipulations, with the following antibiotic concentrations used for selection of strains bearing plasmids or chromosomal resistance cassettes: erythromycin (3 μg/mL), chloramphenicol (5 μg/mL), tetracycline (4 μg/mL). For characterization of growth phenotypes, *S. aureus *strains were grown in Tris-minimal succinate (TMS) [19] broth. TMS culture medium was pretreated with Chelex-100 resin (Bio-Rad) for 24 h at 4°C with 10% (wt/vol) Chelex-100 resin prior to autoclaving. Some micronutrients were added postautoclave. Further culture amendments are detailed below. All media were made with water purified through a Milli-Q water purification system (Millipore, Billerica, MA). All glassware was treated overnight in 0.1 M HCl and rinsed thoroughly with Millipore-filtered water to remove residual contaminating iron.

**Table 1:** Bacterial strains, plasmids, and oligonucleotides used in this study

Reagent	Description	Source or reference
***E. coli***		
DH5α	Φ_80 _dL*acZ*ΔM15 *recA1 endA1 gyrA96 thi*-1 *hsdR17*(r_k_^- ^m_k_^+^) *supE44 relA1 deoR*Δ(*lacZYA-argF*)U169	Promega
***S. aureus ***		
RN4220	r_k_^- ^m_k_^+^; accepts foreign DNA	[20]
RN6390	Prophage-cured wild-type strain	[21]
Newman	Wild-type clinical isolate	[22]
H803	Newman *sirA*::Km; Km^R^	[30]
H1665	Newman Δ*sfa*::Km; Km^R^	[9]
H1666	Newman Δ*sbn*::Tet Δ*sfa*::Km; Tet^R ^Km^R^	[9]
H1262	Newman Δ*hts*::Tet; Tet^R^	[9]
H1497	Newman *sirA*::Km Δ*hts*::Tet; Tet^R ^Km^R^	[9]
H2131	Newman *sbnA*::Tc Δ*sfaABCsfaD*::Km	This study
H1718	Newman *sbnB*::Tc Δ*sfaABCsfaD*::Km	This study
**Plasmids**		
pACYC184	*E. coli *cloning vector; Cm^R^	ATCC
pALC2073	*E. coli/S. aureus *shuttle vector; Ap^R ^Cm^R^	[26]
pAUL-A	Temperature-sensitive *S. aureus *suicide vector; Em^R ^Lc^R^	[25]
pDG1514	pMTL23 derivative carrying tetracycline resistance cassette; Ap^R^	[24]
pFB5	pALC2073 derivative carrying *sbnA*; Cm^R^	This study
pSED52	pALC2073 derivative carrying *sbnB*; Cm^R^	This study
Oligonucleotides*		
Cloning of *sbnA *into pBC SK+		
*sbnA*5'-*Sac*I	5' TGAGCTCGATTCTGTAGGGCAAACACC 3'	
*sbnA*3'-*Kpn*I	5' TTGGTACCTCTAAGTAACGATCGCCTCG 3'	
Amplification/cloning of a tetracycline resistance cassette from pDG1513		
Tet5'-*Nsi*I	5' TTGTATATGCATACGGATTTTATGACCGATGA 3'	
Tet3'	5' TGTGTGGAATTGTGAGCGGATAAC 3'	
Cloning of *sbnA *into pALC2073		
*sbnA*5'-*Xho*I	5' TTTCTCGAGATTTTAAATTTGAGGAGGAA 3'	
*sbnA*3'-*Eco*RI	5' TTTGAATTCCCACATAAACTTGTGAATGATT 3'	
Cloning of *sbnB *into pACYC184		
*sbnB*5'-*Bam*HI	5' TTGGATCCTAGTTTATTCAGATACATGG 3'	
*sbnB*3'-*Bam*HI	5' TTGGATCCTGTCCCAATATTTTGTTGTT 3'	
Cloning of *sbnB *into pALC2073		
*sbnB*5'-*Eco*RI	5' TTGAATTCTCAAGTGATCCATGTAGATG 3'	
*sbnB*3'-*Eco*RI	5' TTGAATTCCAATTCCGGCTATATCTTCA 3'	

* underlined sequences in oligonucleotides denote restriction sites

### DNA and PCR preparation and purification

Plasmid DNA was isolated from bacteria using Qiaprep mini-spin kits (Qiagen), as directed. *S. aureus *cells were incubated for 30 min at 37°C in P1 buffer amended with 50 mg/mL lysostaphin (Roche Diagnostics) prior to addition of lysis buffer P2. Restriction enzymes, T4 DNA ligase, Klenow fragment, and *Pwo*I polymerase were purchased from Roche Diagnostics. *Pfu *Turbo polymerase was purchased from Stratagene and oligonucleotides were purchased from Integrated DNA Technologies. For all PCR reactions, genomic template was from *S. aureus *strain Newman.

### Genetic manipulation and construction of *S. aureus *mutants

All extrachromosomal genetic constructs were created in *E. coli *strain DH5α and then electroporated into the restriction-deficient *S. aureus *strain RN4220 [20] prior to subsequent passage into other *S. aureus *genetic backgrounds. Chromosomal replacement alleles (namely *sbnA*::Tc and *sbnB*::Tc) were generated in strain RN6390 [21] and transduced into the Newman [22] background using phage 80α, similar to previously described methods [9,23]. The *sbnA*::Tc and *sbnB*::Tc mutant alleles and vectors for complementing these mutations *in trans *were generated using methodologies previously described [9,23].

To create an inactivation allele for *sbnA*, the *sbnA *gene was PCR amplified from the chromosome of *S. aureus *strain Newman using primers with engineered *Sac*I and *Kpn*I restriction sites, and cloned into vector pBC SK+. A tetracycline resistance cassette was PCR amplified from vector pDG1514 [24], digested with restriction enzymes *Nsi*I and *Pst*I, and ligated into a unique *Nsi*I restriction site in *sbnA*; this allele was excised and ligated into temperature-sensitive suicide shuttle vector pAUL-A [25] using restriction enzymes *Kpn*I and *Sac*I, then integrated via double homologous recombination into the *S. aureus *RN6390 chromosome. The mutation was transduced to *S. aureus *Newman Δ*sfa *(strain H1665) [9] for use in this study. To generate a complementation vector, *sbnA *was PCR-amplified using primers with engineered *Xho*I and *Eco*RI restriction sites and cloned directly to pALC2073, creating plasmid pFB5.

To create an inactivation allele for *sbnB*, the *sbnB *gene was PCR-amplified from the chromosome of *S. aureus *strain Newman using primers with engineered *Bam*HI sites but cloned as a blunt-ended PCR product to vector pACYC184 digested with *Eco*RV. A tetracycline resistance cassette was excised from vector pDG1514 [24] with restriction enzymes *Nsi*I and *Pst*I and ligated into a unique *Pst*I restriction site in *sbnB *within pACYC184; this allele was excised and ligated into temperature-sensitive suicide shuttle vector pAUL-A using restriction enzyme *Bam*HI, then integrated via double homologous recombination into the *S. aureus *RN6390 chromosome prior to transduction into *S. aureus *Newman Δ*sfa *(strain H1665) for use in this study. To generate a complementation vector, *sbnB *was PCR-amplified using primers with engineered *Eco*RI restriction sites and cloned directly to pALC2073 [26], creating plasmid pSED52.

### Growth assays

*S. aureus *growth curves were generated using a Bioscreen C plate reader (Oy Growth Curves, Finland). Prior to plate inoculation, strains were grown in glass tubes for 12 h in TMS broth and then subcultured and grown for 12 h in TMS broth containing 100 μM 2,2'-dipyridyl (Sigma). Cells were pelleted by centrifugation, washed twice in sterile saline solution, and diluted 1:100 into 200- or 250-μl chelex-treated TMS. Amendments to culture media included 10 μM human holotransferrin (60% iron saturated) (Sigma), 5 mM L- or D-2,3-diaminopropionic acid (Iris Biotech GmbH), 5 mM L-ornithine (Sigma), 5 mM L-alanine, 5 mM *O*-acetyl-L-serine (Sigma), 5 mM L-proline (Sigma), or FeCl_3 _(at 10 or 100 μM). Appropriate antibiotics at the concentrations stated above were included to maintain plasmid selection for complementation experiments. Plates were incubated with constant shaking at medium amplitude. Optical density (OD) was recorded every 15 min, although for graphical clarity, figures have been edited to display values every 2 h.

### Siderophore quantification

Quantification of siderophore output from *S. aureus *strains was performed by testing the iron-binding activity of culture supernatants, using a chrome azurol S (CAS) shuttle solution as described previously [9,27]. Supernatant siderophore units were normalized to culture optical density.

### Siderophore preparations

Siderophore concentrates were prepared by growing *S. aureus *strains with aeration in TMS with 0.1 μM EDDHA. Culture supernatants were harvested at 15 and 40 hours after initial culturing. Cells were pelleted by centrifugation and supernatants were lyophilized. The freeze-dried supernatant was extracted with methanol (one-fifth the original supernatant volume), and then passed through a Whatman No. 1 filter paper to remove insoluble material followed by rotary evaporation. The methanol-extracted material was solubilized in water to 5% of the original supernatant volume. The resulting preparations were stored at -20°C.

### Siderophore plate-disk diffusion assays

Siderophore growth promotion assays were performed essentially as described [9]. Briefly, *S. aureus *strains were seeded into TMS agar (1 × 10^4 ^cells ml^-1^) containing 10 μM EDDHA. Ten-μL aliquots of culture supernatant concentrates (as prepared above) were added to sterile paper disks which were then placed onto the TMS agar plates. Growth promotion was quantified by measuring the diameter of growth around the disc after 36 h at 37°C.

### Computer analyses

DNA sequence analysis, oligonucleotide primer design and sequence alignments were performed either using programs available through NCBI or using Vector NTI Suite software package (Informax, Bethesda, MD). Graphs were generated using GraphPad Prism 4.0.

## Results

### The *S. aureus sbn *operon contains genes predicted to encode L-Dap biosynthesis enzymes

**Figure 1:**
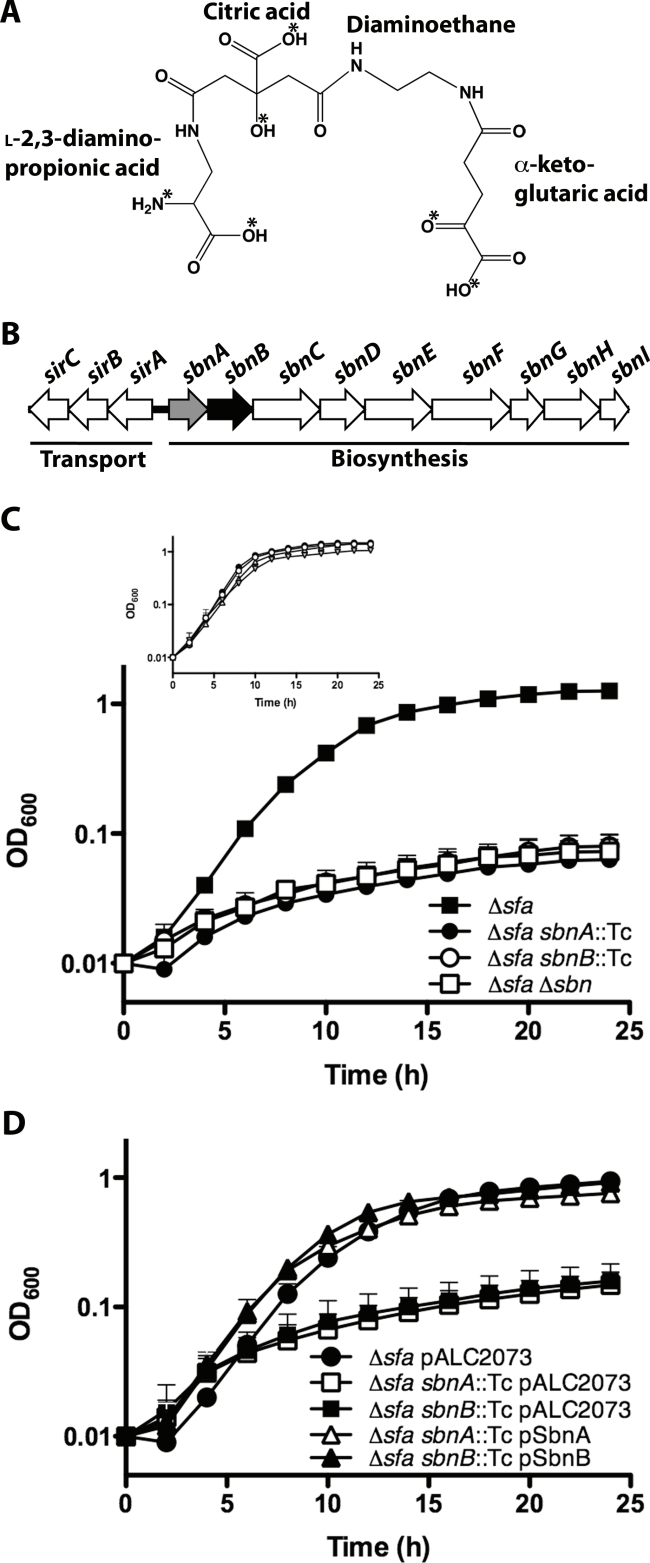
**SbnA and SbnB are essential for synthesis of staphyloferrin B in *S. aureus***. **A) **Chemical structure of staphyloferrin B with fundamental components labeled. Asterisks indicate ligands responsible for the octahedral coordination of iron. **B) **Within the *sir-sbn *genetic locus, the focus of this study is the characterization of mutations in *sbnA *(highlighted grey) (encoding a putative cysteine synthase) and *sbnB *(highlighted in black) (encoding a putative ornithine cyclodeaminase). Together, the products of these two genes are hypothesized to be an L-Dap synthase. **C) ***S. aureus *mutants were grown in chelex 100-treated TMS medium containing 10 μM holo-transferrin. In the Δ*sfa *genetic background, growth in this medium is dependent on the production of the siderophore staphyloferrin B. Supplementation of the medium with FeCl_3 _allows for equivalent growth for all strains (inset). **D) **The growth impairment exhibited by *S. aureus sbnA *or *sbnB *mutants, in the Δ*sfa *genetic background, can be restored upon complementation *in trans *with a wild-type copy of the corresponding gene. Plasmid pALC2073 is the vehicle control.

Original studies on the structural elucidation of staphyloferrin B revealed that it contained citric acid, α-ketoglutaric acid (α-KG), 1,2-diaminoethane (Dae), and L-2,3-diaminopropionic acid (L-Dap) [15] (Figure 1A). The unusual nonproteinogenic amino acid L-Dap serves a critical role for the siderophore in terms of iron-coordination, since a carboxyl group oxygen and the nitrogen atom on the primary amine of L-Dap contribute two of the six iron-ligands used to obtain the distorted octahedral geometry in the ferric-staphyloferrin B complex [28] (Figure 1A). In the proposed biosynthetic pathway, L-Dap is twice incorporated into the staphyloferrin B molecule, as the amine nucleophilic substrate for the type A and type C NIS synthetases SbnE and SbnF, respectively [17]. While SbnE condenses the first molecule of L-Dap to citrate, the action of the decarboxylase SbnH removes the carboxyl group from the L-Dap residue to give rise to the Dae portion of staphyloferrin B [17]. SbnF then condenses a terminal L-Dap onto a citryl-Dae intermediate within the staphyloferrin B structure [17]. Since L-Dap plays such a pivotal role in iron-coordination for staphyloferrin B, and since the biosynthesis of this siderophore requires two units of L-Dap per unit of staphyloferrin B, we were interested in elucidating the genetic requirement for L-Dap biosynthesis in *S. aureus*.

*S. aureus *possesses a nine-gene *sbn *operon with an adjacent *sir *operon; these operons encode proteins that function in staphyloferrin B biosynthesis and transport, respectively [17,23,29,30] (Figure 1B). SbnC, SbnE, SbnF, and SbnH have been previously described as the core enzymes involved in staphyloferrin B biosynthesis [17], however the function of several gene products in the *sbn *operon remain to be resolved. Since L-Dap is such a critical component of staphyloferrin B, we reasoned that the biosynthesis of this molecule must be intrinsic to the *sbn *operon and that L-Dap biosynthesis is likely to occur concurrently with the activity of the rest of the Sbn enzymes. The first two genes in the *sbn *operon are *sbnA *and *sbnB *(Figure 1B) which, through simple NCBI BLAST searches, reveal that they share similarity with cysteine synthases (Table 2) and L-ornithine cyclodeaminases (Table 3), respectively. However, further bioinformatic analyses suggested that genes homologous to *sbnA *and *sbnB *fall under a new family of enzymes currently dubbed "PLP_SbnA_fam" and "dehyd_SbnB_fam", respectively, suggesting that they may carry out functions distinct from the above mentioned enzyme activities. Furthermore, close homologs of these two genes consistently appear adjacent to one another or are genetically fused into a single polypeptide (see Table 4) with the presumed purpose of functioning together to create a biosynthetic precursor. Of particular note are other organisms, in addition to *S. aureus*, that are predicted to produce staphyloferrin B based on the similarity and gene organization of their biosynthetic operons to that of the *S. aureus sbn *operon. Furthermore, genes homologous to *sbnA *and *sbnB *are also found in biosynthetic gene clusters responsible for producing the characterized antibiotics zwittermicin A [31,32], viomycin [18], and dapdiamides [33,34] that all contain L-Dap (Table 2). In support of the presumed role of SbnA and SbnB in L-Dap synthesis, Thomas and colleagues [18] earlier proposed that the enzymes VioB (SbnA homologue) and VioK (SbnB homologue) were involved in production of L-Dap for viomycin synthesis. Therefore, it appears that homologues of *sbnA *and *sbnB *are widely distributed across biosynthetic gene clusters, whose enzymes synthesize molecules for which L-Dap is featured as a structural component.

**Table 2:** List of SbnA homologs

Organism	Similar Protein^a^	PDB ID	Identity (%)	Similarity	E-Value
*Arabidopsis thaliana*	Cysteine synthase	1z7w	33	0.500	0
*Homo sapiens*	Cystathionine beta-synthase	1jbq	30	0.498	0
*Schizosaccharomyces pombe*	Serine racemase	1v71	17	0.202	0
*Escherichia coli*	Biosynthetic threonine deaminase	1tdj	18	0.255	0
*Homo sapiens*	L-serine dehydratase	1p5j	20	0.249	0
*Mycobacterium tuberculosis*	Threonine synthase	2d1f	19	0.279	0
*Pyrococcus furiosus*	Tryptophan synthase beta chain 1	1v8z	22	0.231	0
*Pyrococcus horikoshii*	1-aminocyclopropane-1-carboxylate deaminase	1j0a	19	0.123	0

^a^Only top hit, using HHPred, for each class of enzyme is shown

**Table 3:** List of SbnB homologs

Organism	Homologous Protein^a^	PDB ID	Identity (%)	Similarity	E-Value
*Archaeoglobus fulgidus*	Alanine dehydrogenase	1omo	32	0.543	0
*Homo sapiens*	MU-crystallin homolog	2i99	24	0.381	0
*Pseudomonas putida*	Ornithine cyclodeaminase	1x7d	21	0.352	0
*Thermoplasma volcanium*	Glutamyl-tRNA reductase	3oj0	15	0.312	3e-19
*Geobacillus kaustophilus*	Shikimate 5-dehydrogenase	2egg	13	0.113	1.4e-9

^a^Only top hit, using HHPred, for each class of enzyme is shown

**Table 4:** List of bacteria, not including *S. aureus*, containing transcriptionally-linked *sbnA*/*sbnB *homologs

Organism	Homologous Gene ID (SbnA/SbnB)	Similarity (% SbnA/%SbnB)	E-Value (SbnA/SbnB)	Predicted gene cluster product
*Staphylococcus pseudintermedius *HK10-03	spint_0334/spint_0335	90/91	2e-149/6e-161	Staphyloferrin B
*Cupriavidus metallidurans *CH34	rmet_1117/rmet_1116	75/71	1e-102/2e-97	Staphyloferrin B
*Ralstonia solanacearum *GMT1000	cysK2/rsp0418	76/79	8e-100/2e-118	Staphyloferrin B
*Shewanella denitrificans *OS217	sden_0590/sden_0589	73/75	6e-100/2e-109	Staphyloferrin B
*Methylobacterium nodulans *ORS 2060	mnod_6948/mnod_6949	71/72	9e-91/2e-104	Staphyloferrin B
*Acinetobacter *sp. DR1	aole_07120/aole_07115	76/74	4e-84/6e-104	Staphyloferrin B?**
*Clostridium cellulovorans *743B	clocel_3151/clocel_3150	64/55	4e-65/7e-39	Unknown
*Streptomyces griseus *subsp. Griseus NBRC 13350	sgr_2592/sgr_2591	57/52	1e-56/9e-34	Unknown NRPS product
*Pantoea agglomerans*	*ddaA/ddaB*	56/53	5e-57/3e-32	Dapdiamide antibiotic
*Bacillus thuringiensis *serovar kurstaki YBT-1520	*zwa5A/zwa5B*	63/55	5e-72/3e-48	Zwittermicin A antibiotic
*Streptomyces vinaceus*	*vioB/vioK*	52/47	1e-49/3e-31	Viomycin antibiotic
*Acidobacterium capsulatum *ATCC 51196	acp_1153*	61/44	5e-73/1e-26	Unknown polyketide-NRPS product
*Pseudomonas syringae *pv. tomato DC3000	pspto_2960*	59/49	4e-64/4e-34	Unknown
*Paenibacillus *sp. JDR-2	pjdr2_5192/pjdr_5191	63/49	5e-76/1e-37	Unknown
*Herpetosiphon aurantiacus *ATCC 23779	haur_1863/haur_1864	64/49	8e-77/3e-35	Unknown NRPS product

*Indicates fusion of *sbnA *and *sbnB *homologs into one coding region

**Known gene required for staphyloferrin B biosynthesis (*sbnH *homolog) is missing from operon

### Both *sbnA *and *sbnB *are required for staphyloferrin B biosynthesis in *S. aureus*

The goal of this study was to elucidate the requirement for *sbnA *and *sbnB *in staphyloferrin B synthesis in *S. aureus*, specifically with regard to their presumed role in providing a source of L-Dap in the cell. Under iron-limiting growth conditions, *S. aureus *synthesizes two siderophores, named staphyloferrin A and staphyloferrin B. As we have previously demonstrated, both siderophores play a vital role in acquisition of iron from human holo-transferrin [23]. Moreover, because of functional redundancy, when either the biosynthetic gene cluster for staphyloferrin A (*sfa*) or staphyloferrin B (*sbn*) is inactivated alone (i.e. leaving the other intact in the *S. aureus *cell), the resulting mutants do not display a growth deficit phenotype when human holo-transferrin is provided as the sole iron source. Therefore, the simplest manner in which to study the function of specific genes within the *sbn *operon was to use a strain that was deficient in its ability to synthesize staphyloferrin A; as such, all experiments outlined in this study were performed in a *S. aureus sfa *deletion background.

With holo-transferrin as the sole iron source in the bacterial growth medium, an *S. aureus *Δ*sfa *mutant was capable of growth to an optical density in excess of 1.0 within twenty-four hours (Figure 1C), in agreement with earlier studies [23]. This growth was dependent on an intact *sbn *gene cluster (and, hence, staphyloferrin B production) since the Δ*sfa *Δ*sbn *mutant did not grow above an optical density of 0.1 over the same time period. These growth kinetics were identical to those of *S. aureus *Δ*sfa sbnA*::Tc and *S. aureus *Δ*sfa sbnB*::Tc mutants (Figure 1C), suggesting abrogation of staphyloferrin B production in the absence of either *sbnA *or *sbnB*. Electrospray ionization-mass spectrometry was used to confirm that staphyloferrin B was present in the spent culture supernatant of the Δ*sfa *strain, yet was absent in the spent culture supernatants of the *S. aureus *Δ*sfa sbnA*::Tc and *S. aureus *Δ*sfa sbnB*::Tc strains (data not shown). Importantly, the ESI-MS data were obtained from cultures grown in TMS without added transferrin; this medium is iron-limited but not so much as to completely abrogate growth of siderophore-deficient strains. In order to ensure that the mutant growth deficiencies were not due to pleiotropic effects as a result of the introduction of alternate genetic mutations and that growth, or lack thereof, is solely dependent on iron accessibility, we supplemented each strain with FeCl_3_; this resulted in the growth rescue of all strains (Figure 1C, inset). Moreover, mutants carrying insertions in *sbnA *and *sbnB *were fully complemented by expressing the wild-type version of the genes *in trans*; complemented strains were able to reach a final biomass comparable to the isogenic *S. aureus *Δ*sfa *parental strain (Figure 1D).

### Supplementation of growth media with L-Dap bypasses *sbnA *and *sbnB *mutations, allowing for restored staphyloferrin B production in *S. aureus*

**Figure 2:**
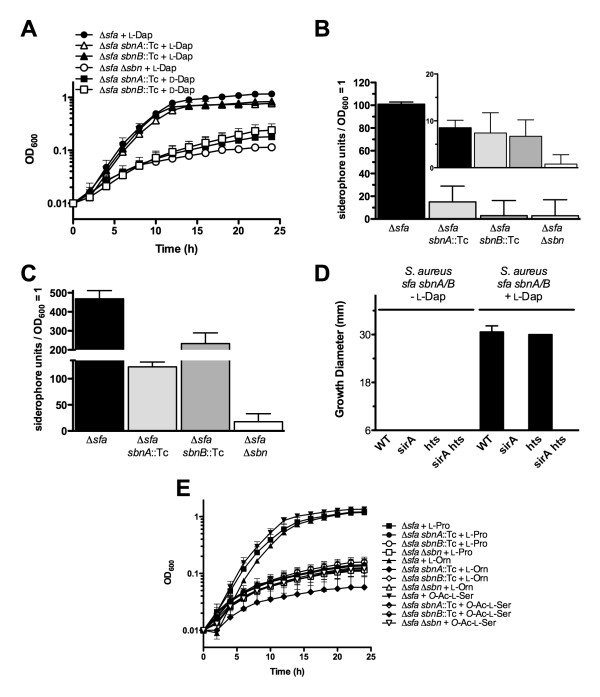
**Supplementation of culture medium with L-Dap allows *S. aureus sbnA *and *sbnB *mutants to overcome the block in synthesis of staphyloferrin B**. **A) **Bacterial growth curves in chelex 100-treated TMS containing 10 μM holo-transferrin as the sole iron source, with the indicated supplements. **B) **Siderophore quantification from culture supernatants of iron-starved *S. aureus *mutants via CAS assay (see Materials and Methods). The inset graph represents culture supernatants from identical strains but grown in medium supplemented with FeCl_3_. Siderophore units are normalized to culture density. **C) **Same as in B) except culture media was supplemented with L-Dap. **D) **Siderophore-disk diffusion assays. Culture supernatants to be tested were derived from *S. aureus *Δ*sfa sbnA*::Tc or Δ*sfa sbnB*::Tc strains cultured in medium supplemented with, or without, L-Dap, as indicated, and were spotted onto sterile paper disks before being placed onto TMS agar plates seeded with *S. aureus *wild-type and siderophore transport mutants, as indicated. Plate disk bioassay is described in Materials and Methods. **E) **Bacterial growth curves for cultures of *S. aureus *Δ*sfa sbnA*::Tc and *S. aureus *Δ*sfa sbnB*::Tc mutants in chelex 100-treated TMS medium containing 10 μM holo-transferrin as the sole iron source, where the medium was supplemented with various hypothesized L-Dap synthase substrates and by-products from Fig. 3, scheme A.

If SbnA and SbnB are involved in the production of L-Dap for staphyloferrin B biosynthesis, then the growth deficit phenotype displayed by *S. aureus *Δ*sfa sbnA*::Tc and *S. aureus *Δ*sfa sbnB*::Tc mutants (Figure 1) should be restored when L-Dap is supplemented in the culture medium, since presence of this molecule would bypass the need for the activities of SbnA or SbnB in siderophore production. Accordingly, as shown in Figure 2A, the iron-restricted growth of *sbnA *and *sbnB *mutants is restored equivalent to that of staphyloferrin B-producing cells when the culture medium of the *sbnA *and *sbnB *mutants is supplemented with L-Dap, but not D-Dap. This is in agreement with the fact that only the L-isomer of Dap is present in the final structure of the staphyloferrin B molecule [15,16,28]. Providing L-Dap to the complete staphyloferrin-deficient mutant (Δ*sfa *Δ*sbn*) did not allow iron-restricted growth, suggesting that growth restoration of *sbnA *and *sbnB *mutants by L-Dap is a result of this precursor being incorporated into a functional siderophore in the presence of other staphyloferrin B biosynthesis enzymes (Figure 2A). This result shows that provision of L-Dap to either *sbnA *or *sbnB *mutants allowed the bypass of the requirement for these genes in staphyloferrin B biosynthesis, which strongly supports the hypothesis that *sbnA *and *sbnB *function together in a direct role in L-Dap synthesis.

Since the iron-restricted growth of *S. aureus *Δ*sfa sbnA*::Tc and *S. aureus *Δ*sfa sbnB*::Tc mutants was restored in the presence of L-Dap, we hypothesized that this was due to the mutants' renewed ability to synthesize staphyloferrin B. To verify this, we performed a chrome azurol S (CAS) assay on concentrated and methanol-extracted culture supernatants of several mutant derivatives of *S. aureus *Δ*sfa *(grown under iron starvation) to quantify their siderophore production (Figure 2B and 2C). Consistent with the growth phenotype illustrated in Figure 2A, amendment of growth media with L-Dap allowed siderophore production by *S. aureus *Δ*sfa sbnA*::Tc and Δ*sfa sbnB*::Tc (Figure 2C). Interestingly, supplementation of the parental strain (Δ*sfa*) with L-Dap enhanced the level of staphyloferrin B output by approximately five-fold (Figure 2C cf. Figure 2B).

**Figure 3:**
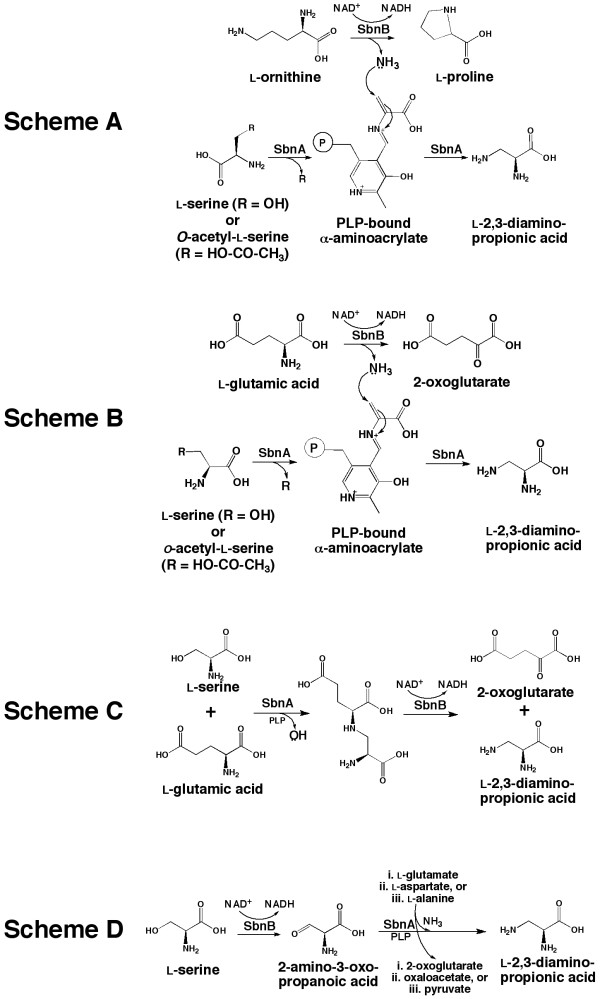
**Proposed schemes for SbnA- and SbnB-dependent synthesis of L-Dap**. Scheme A is adapted from Thomas *et al*. [18] for which the functions of SbnA and SbnB are analogous to the proposed functions VioB and VioK, respectively. The proposed functions of SbnA in schemes B-D remain as a β-replacement enzyme while SbnB is proposed to be an NAD^+^-dependent dehydrogenase of the indicated amino acid.

As a final method to demonstrate that the siderophore secreted by *S. aureus *Δ*sfa sbnA*::Tc or Δ*sfa sbnB*::Tc mutants, in media supplemented with L-Dap, was indeed staphyloferrin B, we performed plate-disk growth promotion assays by spotting culture supernatants onto sterile paper disks that were then placed onto TMS agar seeded with various *S. aureus *siderophore transport mutants (Figure 2D). Only culture supernatants from *S. aureus sbnA*::Tc or *sbnB*::Tc mutants that were fed L-Dap promoted the growth of seeded *S. aureus *Δ*hts *and its isogenic wild-type strain, but strains containing a mutation in the *sirA *gene (encoding the receptor lipoprotein for staphyloferrin B) did not grow. Moreover, no growth-promoting siderophore was produced by *sbnA *or *sbnB *mutants grown in media lacking L-Dap (Figure 2D). LC-ESI-MS/MS was used for confirmation of staphyloferrin B presence in methanol-extracted culture supernatants of complemented mutants (data not shown); spectra were as published previously [17]. When iron-restricted growth media were supplemented with several other molecules that were predicted substrates or byproducts of an SbnA-SbnB reaction (e.g. L-ornithine, L-proline, and *O*-acetyl-L-serine) according to the models illustrated in Figure 3, scheme A, we noted that none rescued the iron-restricted growth of *sbnA *or *sbnB *mutants in the Δ*sfa *background (Figure 2E). This leads us to conclude that none of these molecules can be modified into L-Dap by alternative *S. aureus *enzymes.

## Discussion

In this study we have demonstrated, through a series of genetics-based experiments, that the *S. aureus sbnA *and *sbnB *genes are necessary for staphyloferrin B production. We have also shown that *S. aureus *mutations in *sbnA *and *sbnB *are fully complementable *in trans *by both wild-type copies of each gene as well as through feeding of the molecule L-Dap itself, leading to the renewed production of the staphyloferrin B molecule. The data support the contention that the enzymes SbnA and SbnB function synergistically as a L-Dap synthase, catalyzing the first committed biosynthetic step towards staphyloferrin B synthesis in *S. aureus*. Overall, this is the first study that simultaneously investigates the roles of both genes encoding a cohesive L-Dap synthase.

The L-Dap molecule is a very unusual and rare amino acid. It is non-proteinogenic but it is often found structurally associated with secondary metabolites such as antibiotics (Table 4). To our knowledge, staphyloferrin B represents the only characterized siderophore that contains L-Dap as part of its structure (Figure 1A). The experiment shown in Figure 2A also reinforces the fact that only L-Dap, and not D-Dap, is incorporated into staphyloferrin B. This is in agreement with initial structural elucidation studies [15], the high resolution crystal structure of the siderophore [28], as well as enzymatic recognition of L-Dap as a substrate by staphyloferrin B NIS synthetases [17]. The only siderophore with a component similar to L-Dap in its structure is achromobactin from *Pseudomonas syringae *[35], which has an overall structure and biosynthetic pathway that is very similar to that of staphyloferrin B. In place of L-Dap, achromobactin contains L-2,4-diaminobutyric acid which is condensed onto a unit of citrate and α-KG at both amino groups. L-2,4-diaminobutyric acid may be synthesized by a putative aminotransferase (AcsF) that is also encoded within the achromobactin biosynthetic gene cluster. In the case of achromobactin, synthesis of this diamino acid substrate requires only one enzyme as opposed to the two enzymes required for synthesis of L-Dap. Biochemical characterization of AcsF, along with its substrate specificity, awaits further investigation. Why some siderophore biosynthetic systems have evolved to select one diamino acid over another is an intriguing biological question.

Based on bioinformatics and the emerging diversity of members of the OCD enzyme family, the *S. aureus *SbnB enzyme likely does not contribute to proline production, and hence would not recognize L-ornithine as a substrate. In agreement with this hypothesis, under the experimental conditions of Li *et al*. [36] in testing *S. aureus *for proline synthesis, they could not attribute the production of L-proline to SbnB, but rather L-proline production was solely dependent on the pyrroline-5-carboxylate reductase pathway; it should be noted, however, that these experiments did not test whether *sbnB *was responsible for proline synthesis under low-iron growth conditions and thus do not completely eliminate the possibility that SbnB has OCD activity. The exact biochemical reactions catalyzed by SbnA and SbnB (and homologs) await detailed investigation.

SbnA and SbnB are likely functioning together as an L-Dap synthase and perhaps the mechanism is that originally proposed by Thomas and colleagues [18] for VioB and VioK with regards to viomycin biosynthesis in *Streptomyce*s (Figure 3, scheme A). In this scheme for L-Dap synthesis, VioK (or SbnB) acts as an L-ornithine cyclodeaminase (based on sequence similarity to an OCD [1X7D]) that will convert L-Orn to L-Pro with the concomitant release of ammonia. The released ammonia is picked up by VioB (or SbnA) to be used as a nucleophile for the β-replacement reaction on (O-acetyl-) L-serine, thus generating L-Dap. The reaction catalyzed by VioB (or SbnA) is modeled after homologous cysteine synthases which use a sulfide group for β-replacement reactions to generate cysteine [18]. Therefore, the action of VioB, or SbnA, would appear to be an amidotransferase in this reaction scheme.

However, more recent bioinformatic and phylogenetic analyses of these enzymes suggest that the mechanism of L-Dap synthesis may be quite different from that just described. This is especially true for SbnB, which is more closely related to NAD^+^-dependent amino acid dehydrogenases rather than characterized ornithine cyclodeaminases. Therefore, this prompted us to propose several new mechanisms of L-Dap synthesis (Figure 3, Schemes B-D), emphasizing the role of SbnB as an amino acid dehydrogenase, while SbnA would continue to serve the function of a β-replacement enzyme or aminotransferase. As illustrated in Figure 3, scheme B, SbnB acts as an NAD^+^-dependent L-Glu dehydrogenase that converts L-Glu to 2-oxoglutarate (or α-KG). This reaction will release an ammonia molecule to be used by SbnA in an identical manner to the second half of the reaction proposed in scheme A. The reaction depicted in scheme B is attractive since all products of this mechanism can be funneled towards staphyloferrin B biosynthesis (i.e. α-KG is a substrate for SbnC, while L-Dap is a substrate for SbnE and SbnF), as opposed to scheme A where the generation of L-Pro serves no purpose in staphyloferrin B biosynthesis. In scheme C, SbnA would act as the first enzyme in the pathway by condensing L-Ser with L-Glu to form a larger intermediate consisting of an L-Ser-L-Glu conjugate. In effect, SbnA would perform a β-replacement reaction on L-Ser by displacing the hydroxyl group on L-Ser with L-Glu. Dehydrogenase activity provided by SbnB would resolve and split the intermediate compound to give rise to L-Dap and 2-oxoglutarate. As in scheme B, all products from this reaction are used in the biosynthesis of staphyloferrin B. In scheme D, SbnB would serve as a 2-Ser dehydrogenase, converting L-Ser to 2-amino-3-oxopropanoic acid, an intermediate that would be primed for nucleophilic attack at the β-carbon by an ammonia molecule derived from the aminotransferase activity of SbnA. The source of this ammonia might be L-Glu, L-Asp, or L-Ala, which are all common amino group donors. In this scheme, if L-Glu is used as the amino donor, 2-oxoglutarate is produced and would be a substrate for the SbnC synthetase.

Unlike the substrate uncertainty exhibited by SbnB, the substrate for SbnA homologs have been defined through precursor labeling studies [37,38]. Since, an SbnA homologue is involved in L-Dap production for viomycin (Table 4), then it is very likely that L-serine (or the *O*-acetylated derivative) is also the substrate for SbnA. Moreover, a recent study by Zhao *et al*. [32] characterized the gene *zwa5A*, an SbnA homologue (Table 4), and through genetic knockout of this gene in *Bacillus thuringiensis*, confirmed that it is involved in synthesizing L-Dap for the antibiotic zwittermicin A. Similar to our experiments, these researchers were able to restore the production of zwittermicin A in the *zwa5A *mutant by providing exogenous L-Dap to the culture media [32].

It is important to note that β-replacement reactions involving ammonia as the nucleophile are rare. Only recently was an L-2,3-diaminobutyric acid (L-Dab) synthase studied that is involved in mureidomycin A production [39]. This enzyme, which catalyzes a similar reaction to the ones proposed for SbnA (Figure 3), will use L-Thr as the substrate (instead of L-Ser) and will displace the β-hydroxyl group with an ammonia molecule to form L-Dab. However, the source of the ammonia was not described and thus it is assumed that this enzyme may depend on cellular concentrations of free ammonia rather then receiving the ammonia from a dedicated dehydrogenase. The idea that an enzyme acquires free ammonia within a cell is intriguing. Certainly, the rate of diffusion of ammonia inside a cell can be a limiting factor and this is perhaps why both halves of an L-Dap synthase appear to be consistently co-expressed, and potentially are intimately associated with one another such that liberated ammonia by the dehydrogenase unit can be properly channeled to the aminotransferase unit. This would ensure catalytic efficiency and also assumes that extensive protein-protein interactions would occur between the two enzymes. Certainly, this idea is supported by the existence of single-polypeptide encoding genes found within the *P. syringae *and *Acidobacterium capsulatum *genome, in which half of the polypeptide shares significant similarity with SbnA and the other half shares significant similarity with SbnB (Table 4).

It is interesting that supplementation of the *S. aureus *culture medium with L-Dap enhanced staphyloferrin B output in wildtype cells (Figure 2B cf. 2C), a phenomenon that has previously been observed [15]. It is tempting to speculate that L-Dap may be a critical molecule in terms of regulating staphyloferrin B production or that the presence of L-Dap is a signal for the organism to commit to staphyloferrin B synthesis. At this time, how such a signal may be transduced is unknown but perhaps L-Dap can induce allosteric effects on certain Sbn proteins that may, in turn, regulate staphyloferrin B production. Interestingly, enhancement of end product formation by L-Dap feeding has also been observed for zwittermicin A production in *B. thuringiensis *[32].

The biochemical schemes for L-Dap synthesis, as depicted in Figure 3, await experimentation with purified enzymes as well as screening with potential substrates, and these experiments are under investigation in our laboratory. Certainly, the actual mechanism of L-Dap synthesis may not be restricted to those mechanisms outlined here, but at least these provide a starting point towards the biochemical investigation of L-Dap synthase enzymes in different bacteria. No matter the mechanism, it is most surely to be novel. Regardless, the studies here have demonstrated the essentiality of SbnA and SbnB towards L-Dap synthesis in *S. aureus*, a nonproteinogenic amino acid component of staphyloferrin B that is critical to the iron coordinating function of the siderophore, as well as providing implications for the role that L-Dap may play in regulating production of the molecule.

## Conclusions

Mutation of either *sbnA *or *sbnB *result in abrogation of synthesis of staphyloferrin B, a siderophore that contributes to iron-restricted growth of *S. aureus*. The loss of staphyloferrin B synthesis is due to an inability to synthesize the unusual amino acid L-2,3-diaminopropionic acid which is an important, iron-liganding component of the siderophore structure. It is proposed that SbnA and SbnB function together as an L-Dap synthase in the *S. aureus *cell.

## Authors' contributions

FCB and JC carried out the molecular genetic and bioinformatics studies and drafted the manuscript. All authors participated in the design of the study, and edited and approved the final version of the manuscript.
